# Therapeutic Agency of Mesmerism

**Published:** 1844-11

**Authors:** 


*Therapeutic Agency of Mesmerism.*—Mention is made in a New Orleans paper of a young lady who, during the mesmeric sleep, had a tooth extracted, without being conscious of any pain. The fact was very astonishing, till it was subsequently ascertained that she had had *sixty-two extracted* before public audiences, to illustrate the wonderful effects of the new science!

*Boston Medical and Surgical Journal.*

